# Ag_2_S-Decorated One-Dimensional CdS Nanorods for Rapid Detection and Effective Discrimination of n-Butanol

**DOI:** 10.3390/nano14050394

**Published:** 2024-02-21

**Authors:** Yubing Gao, Weirong Zhou, Yong Wang, Yuan Gao, Jiayin Han, Dehao Kong, Geyu Lu

**Affiliations:** State Key Laboratory on Integrated Optoelectronics, Key Laboratory of Gas Sensors, College of Electronic Science and Engineering, Jilin University, 2699 Qianjin Street, Changchun 130012, China; gaoyb19@mails.jlu.edu.cn (Y.G.); zhouwr21@mails.jlu.edu.cn (W.Z.); yongw22@mails.jlu.edu.cn (Y.W.); hanjy22@mails.jlu.edu.cn (J.H.); kongdh21@mails.jlu.edu.cn (D.K.); lugy@jlu.edu.cn (G.L.)

**Keywords:** CdS, Ag_2_S, n-butanol, heterostructure, gas sensor

## Abstract

N-butanol (C_4_H_9_OH) is a volatile organic compound (VOC) that is susceptible to industrial explosions. It has become imperative to develop n-butanol sensors with high selectivity and fast response and recovery kinetics. CdS/Ag_2_S composite nanomaterials were designed and prepared by the solvothermal method. The incorporation of Ag_2_S engendered a notable augmentation in specific surface area and a consequential narrow band gap. The CdS/Ag_2_S-based sensor with 3% molar ratio of Ag_2_S, operating at 200 °C, demonstrated a remarkably elevated response (S = R_a_/R_g_ = 24.5) when exposed to 100 ppm n-butanol, surpassing the pristine CdS by a factor of approximately four. Furthermore, this sensor exhibited notably shortened response and recovery times, at a mere 4 s and 1 s, respectively. These improvements were ascribed to the one-dimensional single-crystal nanorod structure of CdS, which provided an effective path for expedited electron transport along its axial dimension. Additionally, the electron and chemical sensitization effects resulting from the modification with precious metal sulfides Ag_2_S were the primary reasons for enhancing the sensor response. This work can contribute to mitigating the safety risks associated with the use of n-butanol in industrial processes.

## 1. Introduction

N-butanol has been found extensive application in the industrial field as a biochemistry extractant and surfactant. Even minuscule concentrations of n-butanol can emit a pungent odor, while concurrently inducing deleterious physiological effects, encompassing anesthesia, cephalalgia, and corneal damage. Moreover, at ambient concentrations of n-butanol vapor ranging from 1.45% to 11.25%, there is a clear risk of accidents such as combustion or explosion when the temperature exceeds its flash point, posing a serious threat to the safety of human life and industrial production [1,2,3]. To ensure the utmost safety and health for practitioners in production, the permissible concentration of n-butanol in the workplace is limited to below 150 mg/m^3^ (~45 ppm). Consequently, the development of n-butanol gas sensors with expeditious response and recovery kinetics, exceptional selectivity, and enduring stability assumes profound significance.

Semiconductor gas sensors represent a highly ubiquitous and utilitarian category of gas sensors. Among them, chemical resistive gas sensors, due to their simplicity in structure, ease of manufacture, and superior sensing performance, have attracted extensive attention [4,5]. Compared with conventional metal oxide semiconductors, devices utilizing metal sulfides as sensitive materials often exhibit shorter response or recovery times. CdS is a crucial n-type semiconductor material, which is widely applied in various fields, such as fluorescence labeling, photocatalysis, solar cells, and optoelectronic devices [6,7,8]. It can be synthesized using various methods, including solution-based techniques, vapor deposition, and thermal decomposition [9,10,11]. This versatility enables relatively easy synthesis of CdS and allows for control over its morphology and crystal structure, facilitating the desired performance optimization [12,13,14]. In recent years, nanostructured CdS materials have attracted significant interest from researchers as gas-sensing substrates. Studies in the literature have shown that CdS demonstrates rapid response and recovery characteristics to alcohol gases, making it a promising advanced functional material for VOC gas detection. For instance, Feng et al. have synthesized CdS nanospheres doped with graphene, demonstrating exceptional selectivity and a fast response and recovery speed to ethanol [15]; Chakraborty et al. reported that CdS nanoflakes prepared in their study showed fascinating performance for ethanol (response 30% to 1 ppm) [16]; Xue et al. have developed a nano-CdS sensor with the capability to detect alcohols [17]; the CdS nanocrystal prepared by Bu et al. exhibited a response value exceeding 40 to n-propanol, with a response time of less than 1 s [18]; Ma et al. reported that the sensor based on Au nanoparticle-decorated CdS nanowires showed a high response to ethanol (approximately 110 to 100 ppm) and swift response and recovery kinetics [8]. Therefore, we aim to further investigate the gas sensing properties of CdS towards n-butanol. 

Precious metals and their derivatives usually exhibit catalytic properties, which are used to modify the matrix material for further improvement of sensing properties. Ag_2_S is a semiconductor precious metal sulfide with a narrow bandgap (~1 eV), excellent chemical stability, and exceptional electronic characteristics. It has been applied in the field of optoelectronic devices, and has also drawn recent attention in the realm of gas sensing applications [19]. Shafi et al. demonstrated that doping Ag_2_S into NiO-ZnO heterojunction composites improved the sensitivity to acetone by generating more catalytic sites and increasing electron transfer rates [20]; A-Rang Jang et al. achieved room-temperature detection of acetone by decorating graphene with Ag_2_S [19]; Fu et al. developed an innovative ammonia gas sensor based on Ag_2_S film, which exhibited a response value of 297 to 7.08% ammonia at a working voltage of 2 V [21]; Wang et al. designed an Ag_2_S/SnS_2_ heterojunction for fast detection of NO_2_ at a concentration as low as 1 ppm [22]. Therefore, integrating Ag_2_S to construct composite materials emerged as an efficient approach to enhance gas sensitivity and expedite response and recovery rates.

In this study, a one-step solvothermal technique was employed to synthesize CdS single-crystal nanorods, decorated with various contents of Ag_2_S. The effect of Ag_2_S content on the morphology, bandgap, work function, etc., were systematically investigated. Furthermore, the sensing properties to n-butanol were comprehensively assessed, including selectivity, response/recovery characteristics, repeatability, humidity resistance, and long-term stability of the sensor. The associated mechanism would be elucidated, focusing on the morphology control and modification with precious metal sulfide. The high performance of this sensor substantiated the developmental potential of CdS as a sensitive material in the field of n-butanol detection.

## 2. Experimental

### 2.1. Material Synthesis

Preparations of 3 mmol cadmium acetate (Cd (Ac)_2_·2H_2_O), 9 mmol thiourea (CH_4_N_2_S), and AgNO_3_ with different molar ratios (Ag:Cd = 0%, 1%, 3%, and 5%) were added to a beaker containing 10 mL deionized water and 20 mL C_2_H_8_N_2_. The mixture was vigorously stirred at room temperature until complete dissolution, resulting in the formation of a homogeneous solution. Subsequently, this solution was transferred into a 50 mL Teflon-lined stainless steel autoclave, sealed, and subjected to heating at 175 °C for 8 h. Following the completion of the reaction, the solution was allowed to naturally cool to room temperature. The resultant product was subjected to multiple cycles of centrifugation with deionized water and ethanol and subsequently dried in a vacuum environment at 60 °C for a duration of 12 h. The final products were named as pristine CdS, ACS-1, ACS-3, and ACS-5, respectively.

### 2.2. Characterization

The microstructure, lattice pattern, and surface element distribution of the sensing material were observed using scanning electron microscopy (SEM, JSM-7500 F, 10 kV, Japan Electronics Co., Ltd., Tokyo, Japan), transmission electron microscopy and energy-dispersive X-ray spectroscopy (TEM and EDS, JEOL JEM-2100 F, 200 kV, Japan Electronics Co., Ltd., Tokyo, Japan). The chemical composition of the sensing material was characterized by X-ray diffraction (XRD, Rigaku D/max-2550, Rigaku Corporation, Tokyo, Japan, Cu Kα1 radiation with λ = 1.54 Å, X-ray: 40 kv, 200 mA, scan step: 0.02°). The surface chemical state of the sensing material was determined by X-ray photoelectron spectroscopy (XPS, Thermo Scientifific K-Alpha, Waltham, MA, USA, ESCALABMKII, Mg Kα radiation with hν = 1253.6 eV). The light absorption capacity and bandgap width of the sensing material were measured by UV-visible spectroscopy (UV-vis, Shimadzu UV-2550, Shimadzu Corporation, Kyoto, Japan). The work function of the sensing material was measured by Kelvin probe force microscopy (KPFM, KP 6500 Digital Kelvin Probe McAllister Technical Services Co., Ltd., Coeur d’Alene, ID, USA). The specific surface area of the sensing material was tested by Brunauer–Emmett–Teller (BET, Micromeritics Gemini VII, Norcross, GA, USA) analysis.

### 2.3. Sensor Fabrication and Gas Sensing Measurements

A 10 mg quantity of sample powder was blended in a mortar with 2 drops of deionized water to create a homogeneous paste. Subsequently, the resultant paste was uniformly and comprehensively applied onto both the external surface of Al_2_O_3_ tube and gold electrodes using a brush. The length of the ceramic tube is 6 mm, and the outer diameter is 1.2 mm. Therefore, the total effective area exposed to the gas is approximately 22.6 mm^2^. And the thickness of the active layer is typically around 20–30 μm. The nichrome heating coil was then meticulously threaded through the interior of the ceramic tube. Ultimately, four Pt pins of the Al_2_O_3_ tube and the two terminations of the nichrome heating coil were soldered to the hexagonal base of the sensor.

The gas sensing test system and the testing steps are the same as in our previous work [23]. The system for evaluating gas sensing performance comprises integral components, including a computer, testing chambers, a precision constant current source (GPD-3303S, Instek Digital, New Taipei City, Taiwan), and a FLUKE 8864A tester (Fluke Corporation, Everett, WA, USA). The testing protocol is briefly described as follows: Initially, the sensor was stabilized within a chamber filled with ambient air as background. Then, the device was transferred to a chamber containing the target gas, and the responsive resistance was recorded until achieving equilibrium. Subsequently, the sensor was placed back into the air-filled chamber to complete the recovery process. The constant current source was employed to regulate the output current, thereby modulating the operational temperature of the gas sensor. In this work, the response to reducing gases was defined as S = R_a_/R_g_, where R_a_ and R_g_ represent the stable resistance values of the sensor in air and target gas, respectively. For oxidative gases, the response of the sensor was defined as S = R_g_/R_a_. The response and recovery time were defined as the duration necessary for the resistance change to reach 90% upon introduction or removal of the target gas, respectively.

## 3. Results and Discussions

### 3.1. Material Characterization

**Figure 1:**
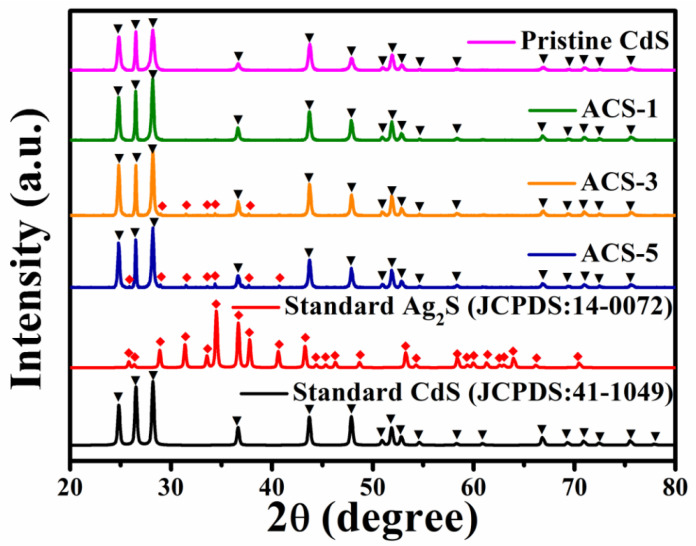
XRD patterns of pristine CdS and CdS/Ag_2_S. ▼ and ◆ indicate diffraction peaks belonging to CdS and Ag_2_S, respectively.

Figure 1 illustrates the XRD patterns of the pristine CdS and CdS/Ag_2_S composite materials with different molar ratios. The pattern of pristine CdS demonstrates a strong concordance with the hexagonal CdS standard card, exhibiting narrow and sharp peak profiles without any observed superfluous peaks. This revealed the high purity and excellent crystallinity of the sample. With the increase in Ag_2_S content, peaks matching with the monoclinic Ag_2_S standard card (JCPDS: 14-0072) are gradually observed. The peaks corresponding to Ag_2_S were not observed in ACS-1, possibly due to the tiny content of Ag_2_S. It is noteworthy that the position and configuration of the diffraction peaks attributed to CdS in all samples were not observed with significant variation, indicating that the one-step solvothermal method employed for introducing Ag_2_S has no discernible impact on the crystal structure of CdS.

**Figure 2:**
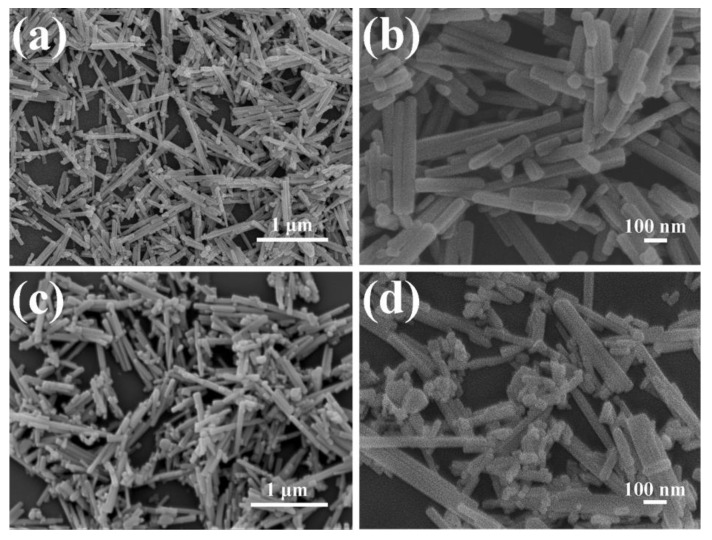
Low- and high-resolution SEM images of (**a**,**b**) pristine CdS and (**c**,**d**) ACS-3.

The sample powders were deposited on the silicon substrate for FESEM observation, which images are presented in Figure 2 and Figure S1. The pristine CdS sample exhibits a smooth rod-like morphology with a consistent cross-sectional diameter ranging from 50 to 60 nm and a longitudinal length spanning 50 to 500 nm. The introduction of Ag_2_S did not cause a considerable change in the size of the CdS rod-like structure. Some irregular Ag_2_S particles can be observed deposited on the surface of CdS, and a small amount of Ag_2_S particles aggregated. Additional and larger-sized Ag_2_S particles can be clearly observed with the increase in Ag_2_S content. Furthermore, as shown in Figure S2c,d, a denser aggregation of CdS nanorods is observed surrounding the bulk Ag_2_S.

**Figure 3:**
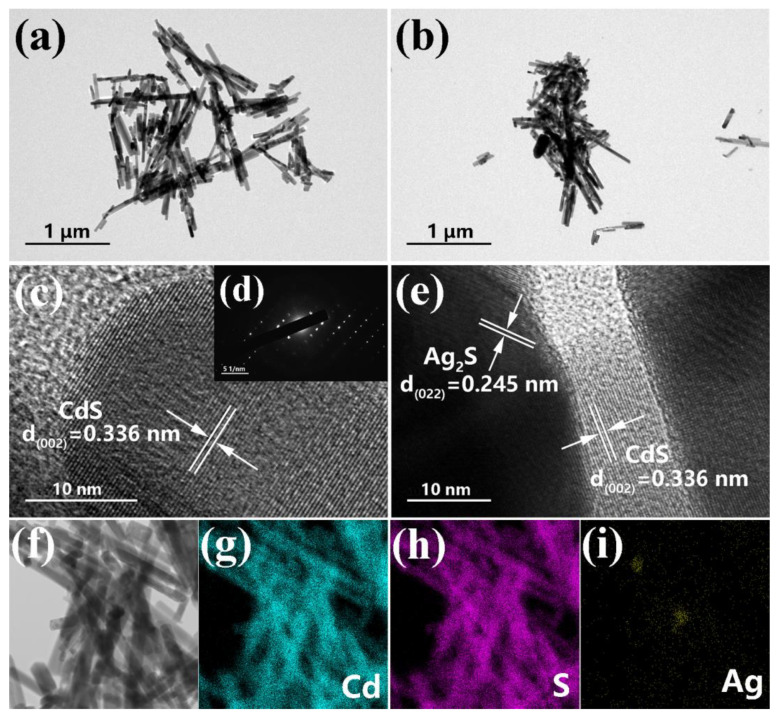
TEM images of (**a**) pristine CdS and (**b**) ACS-3; HRTEM images of (**c**) pristine CdS and (**e**) ACS-3; (**d**) SAED patterns of ACS-3; (**f**) STEM image and (**g**–**i**) element mapping images of ACS-3.

TEM was utilized to obtain further information about the structure and surface elements. Figure 3a,b display the TEM images of pristine CdS and ACS-3. The rod-shaped structure described in the SEM images and irregular Ag_2_S nanoparticles adhering the nanorods in ACS-3 were discernible. In the TEM image of ACS-1 (Figure S3a), it is challenging to observe Ag_2_S particles. However, in the TEM image of ACS-5 (Figure S3b), larger-sized irregular Ag_2_S particles can be observed, which is consistent with the SEM characterization results. In the HRTEM images shown in Figure 3c–e, ordered lattice fringes spacings of 0.336 and 0.245 nm, respectively, correspond to the (002) crystal plane of the hexagonal CdS and the (022) crystal plane of the monoclinic Ag_2_S. The orderly arrangement of diffraction spots depicted in inset (d) indicated that the prepared CdS nanorods are well-grown single crystals. In contrast to polycrystals, electrons in single crystals do not need to cross many grain boundaries, which is conducive to accelerating transmission. To determine the distribution of Ag_2_S nanoparticles, EDS surface scanning was conducted on ACS-3 for the elemental characterization. The results are depicted in Figure 3g–i, where Cd and S elements exhibited distinct nano-rod stacking profiles, while the aggregation of Ag elements provides further evidence of the attachment of Ag_2_S in the form of nanoparticles onto the CdS nanorods.

**Figure 4:**
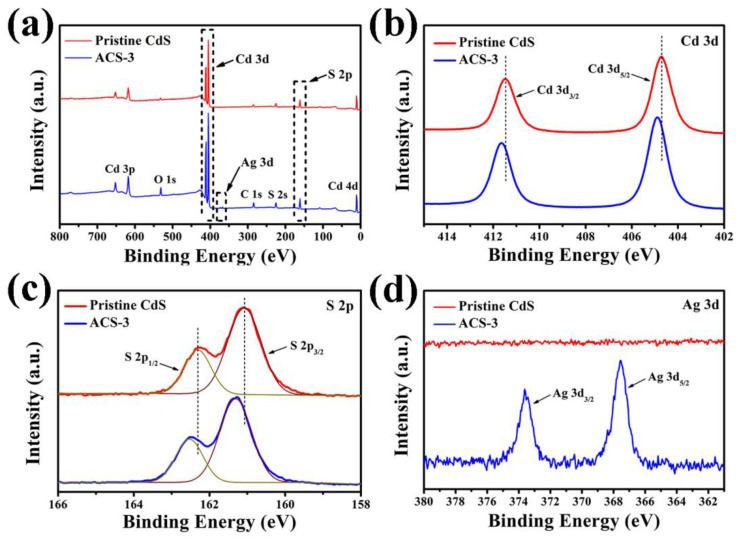
(**a**) XPS survey spectra, (**b**) Cd 3d, (**c**) S 2p, and (**d**) Ag 3d high-resolution spectra of pristine CdS and ACS-3.

The elemental chemical states of pristine CdS and ACS-3 were characterized utilizing XPS, employing C 1s peak as the calibration peak. Figure 4a exhibits the corresponding peaks of Cd, S, Ag, O, and C, indicating the absence of any impurity elements in the sample. The Cd 3d spectrum in Figure 4b displays two peaks, with the positions of the two peaks for pristine CdS at 404.73 and 411.48 eV, respectively, corresponding to Cd 3d_5/2_ and Cd 3d_3/2_ orbitals of Cd^2+^. For ACS-3, the two peaks shift slightly to 404.88 and 411.63 eV, respectively. The S 2p spectrum in Figure 4c shows two deconvoluted peaks for pristine CdS at 161.08 and 162.31 eV in correspondence to S 2p_3/2_ and S 2p_1/2_ orbitals of S^2−^, respectively, while the positions of peaks move to 161.31 and 162.48 eV for ACS-3, respectively. Both Cd 3d and S 2p in ACS-3 are slightly shifted to higher energies compared to pristine CdS, mainly due to the introduction of Ag_2_S, which affects the chemical environment of Cd^2+^ and S^2−^. The peaks at 367.58 and 373.63 eV in Figure 4d were in correspondence to Ag 3d_5/2_ and Ag 3d_3/2_ orbitals of Ag^+^ in ACS-3, respectively, while no peaks belonging to Ag^+^ are observed in pristine CdS. These analysis results confirm the successful fabrication of CdS/Ag_2_S heterojunction [24,25].

**Figure 5:**
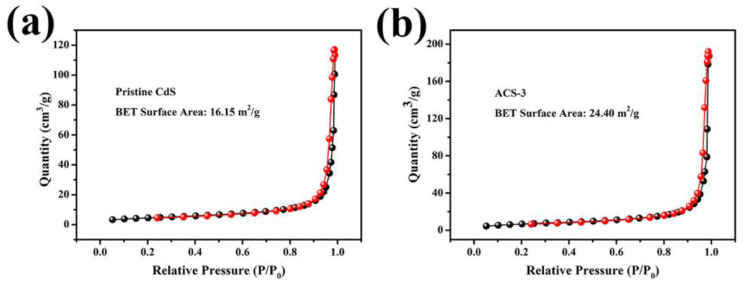
N_2_ adsorption–desorption isotherms of (**a**) pristine CdS and (**b**) ACS-3. Black and red respectively represent the adsorption and desorption curves.

**Figure 6:**
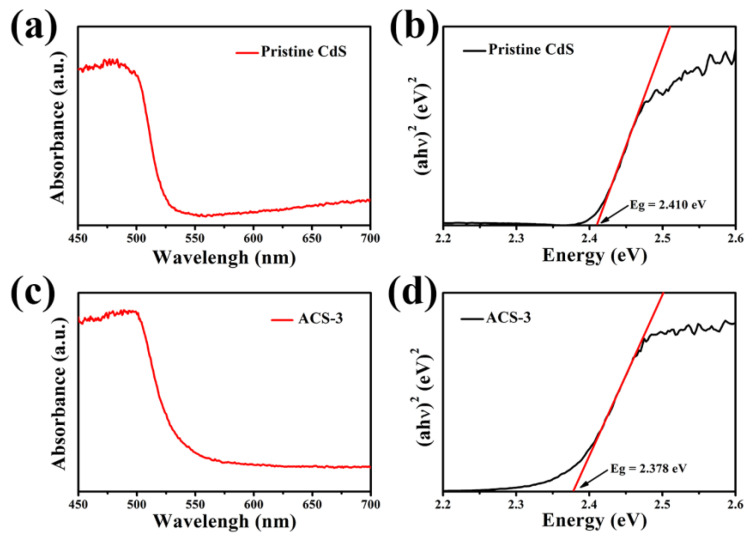
The UV-vis diffuse reflectance spectra of (**a**) pristine CdS and (**c**) ACS-3; bandgap energy approximations of (**b**) pristine CdS and (**d**) ACS-3. The red line in (**b**) or (**d**) is the tangent of the relationship curve between (ahν)^2^ and Energy.

Figure 5 and Figure S4 show the specific surface areas of pristine CdS, ACS-1, ACS-3, and ACS-5, which are 16.15, 18.20, 24.40, and 23.92 m^2^g^−1^, respectively. The specific surface area of ACS-1 and ACS-3 gradually expands compared to pristine CdS, possibly due to the presence of small-sized Ag_2_S nanoparticles in the gaps generated by the stacking of CdS nanorods. However, when Ag_2_S is excessive, the larger-sized Ag_2_S particles lead to a decrease in the specific surface area of ACS-5 compared to ACS-3. Therefore, ACS-3 exhibits the largest specific surface area among these samples. The increase in specific surface area upon introducing Ag_2_S is beneficial for improving gas sensing performance. Further characterization and analysis were conducted to explore the effect of Ag_2_S on the electronic properties of the heterostructure. The UV-Vis diffuse reflectance spectra of all samples are shown in Figure 6 and Figure S5. According to the reported literature, the absorption edge at around 525 nm for pristine CdS can be defined as the intrinsic bandgap absorption of CdS [6]. After introducing Ag_2_S, the light absorption band of the CdS/Ag_2_S composite material showed a slight red shift. The bandgap energy is related to the light absorption characteristics of the sensing material and can be estimated using the Tauc plot [26]:

αhν = B(hν − E_g_)^n^ (1)

The bandgaps of pristine CdS, ACS-1, ACS-3, and ACS-5 were approximately 2.410, 2.398, 2.378, and 2.373 eV. The bandgap of pristine CdS is close to the value of bulk CdS reported in ref [27]. Although the bandgap of materials depends on the versatile synthesis, size, morphology, etc., the bandgap value of Ag_2_S (1~2.26 eV) reported in the literature [28,29] is smaller than our measured value of pristine CdS. Thus, the bandgap narrowing is proposed due to the introduction of narrow-bandgap Ag_2_S and the formation of heterojunctions with CdS. As the content of Ag_2_S increases, the bandgap of the composite nanomaterial gradually narrows. A narrower bandgap contributes to easier electron transitions from the valence band to the conduction band, thus facilitating more carrier participation in the formation process of surface-adsorbed O^−^, which is beneficial for enhancing sensors’ performance [30,31,32].

### 3.2. Sensing Properties Analysis

**Figure 7:**
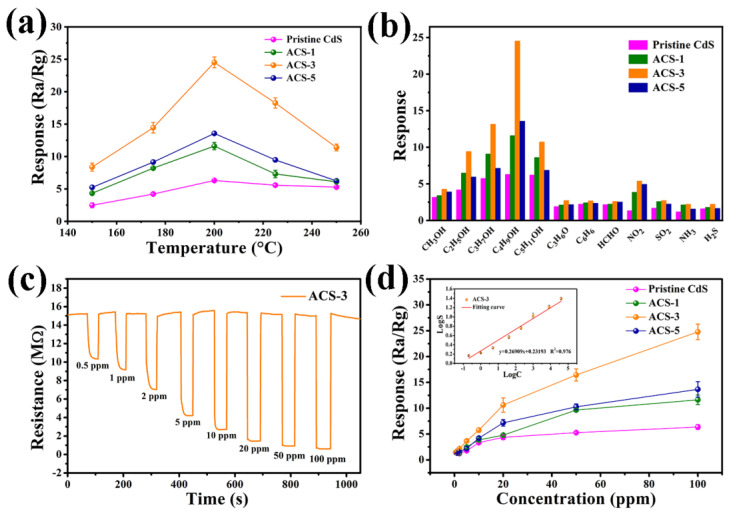
(**a**) Responses of sensors based on all samples at different operating temperature to 100 ppm n-butanol; (**b**) responses of sensors based on all samples to different gases at 200 °C; (**c**) the dynamic response curves of the sensor based on ACS-3 to n-butanol at different concentrations (0.5 ppm~100 ppm) at 200 °C; (**d**) the correlation curve of the response of the sensor with n-butanol concentrations at 200 °C.

The performance of semiconductor metal sulfide gas sensors is greatly affected by the working temperature. Optimal temperature can lead to an elevation in carrier concentration, activation of the sensitive material, and enhancement of surface O^−^ adsorption [33]. Figure 7a illustrates all sensors’ response to 100 ppm n-butanol within 150 °C~250 °C. The response-temperature plots of all sensors exhibit an initial ascending trend followed by a subsequent decline. This phenomenon stems from two factors: Firstly, the increased temperature provides more energy to facilitate the reaction between gas molecules and adsorbed oxygen; Secondly, the heightened thermal motion of gas molecules promotes their desorption. The two factors collectively determine the optimal working temperature [34]. Evidently, all sensors exhibited the maximum response at 200 °C. Moreover, the modification of Ag_2_S significantly enhanced the response at any working temperature within the measured range. ACS-3 demonstrated the highest response (24.5), which exhibited a 3.9-fold enhancement compared to pristine CdS (6.28). However, excessive Ag_2_S tended to agglomerate, thereby reducing the number of active sites and the content of effective heterojunctions. This inhibited the interaction between n-butanol molecules and ACS-5, leading to a decrease in sensitivity.

The response bar charts of all sensors to various gases are depicted in Figure 7b, including 100 ppm methanol, ethanol, n-propanol, n-butanol, n-pentanol, acetone, benzene, formaldehyde; as well as 5 ppm NO_2_, SO_2_, NH_3_, and H_2_S. It is evident that ACS-3 demonstrated superior selectivity towards n-butanol among interfering gases. The distinct intrinsic properties of interfering gases serve as the fundamental reason for the change in selectivity resolution. Notably, it is observed that the sensitivity of all sensors to straight-chain alcohol gases obviously surpassed that of other gases. The relationship between response and the number of carbon atoms can be observed from the column chart. With the increase in carbon atoms, the response shows a trend of initially rising and then declining. The sensor based on ACS-3 demonstrated response values of 4.27, 9.43, 13.14, 24.52, and 10.72 to 100 ppm CH_3_OH, C_2_H_5_OH, C_3_H_7_OH, C_4_H_9_OH, and C_5_H_11_OH, respectively. The sensor exhibits the highest response to n-butanol.

Figure 7c displays the dynamic response–recovery curves of the ACS-3 gas sensor when exposed to n-butanol concentrations spanning 0.5~100 ppm at 200 °C. In Figure 7d, the correlation between sensor responses and n-butanol concentration is depicted. Evidently, all sensors’ responses exhibit an upward trend as the concentration of the target gas increased. However, only the ACS-3 sensor demonstrated the capability to detect n-butanol at a concentration as low as 0.5 ppm, yielding a response value of 1.47. In comparison, the detection limits (corresponding to response values) of the pristine CdS, ACS-1, and ACS-5 sensors were 2 ppm (1.23), 2 ppm (1.31), and 1 ppm (1.32), respectively. Remarkably, the response value of the ACS-3 sensor still showed an upward trend without reaching saturation even at 100 ppm, indicating that this sensor not only had a low detection limit but also a broad detection span for n-butanol. The concentration–response relationship of the ACS-3 sensor towards n-butanol is depicted in the inset of Figure 7d, exhibiting a linear correlation. The correlation equation, LogS = 0.27LogC + 0.23 (R^2^ = 0.976), defines the relationship between the sensor’s response value (S) and the concentration of n-butanol (C).

**Figure 8:**
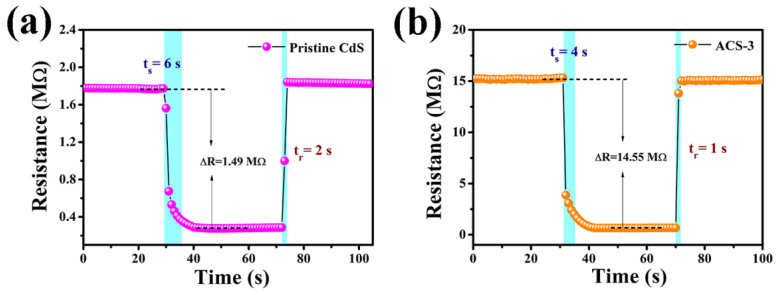
Transient response–recovery curves of the sensors based on (**a**) pristine CdS and (**b**) ACS-3 to 100 ppm n-butanol at 200 °C. The blue areas represent the response−recovery times.

To obtain detailed information about sensing characteristics, dynamic response–recovery curves of the sensors are shown in Figure 8 (200 °C and 50% RH). The response and recovery time for the pristine CdS-based sensor were 6 s and 4 s, respectively, while those for the ACS-3 sensors were 2 s and 1 s, respectively. Both sensors exhibited fast response/recovery characteristics to target gas, with particular emphasis on the nearly instantaneous recovery process upon removal from the n-butanol atmosphere. Table 1 provides a comparative analysis of the gas performance of n-butanol gas sensors employing different sensitive materials. It can be seen that the n-butanol sensor had a notable superiority in response/recovery time at relatively low working temperatures.

**Figure 9:**
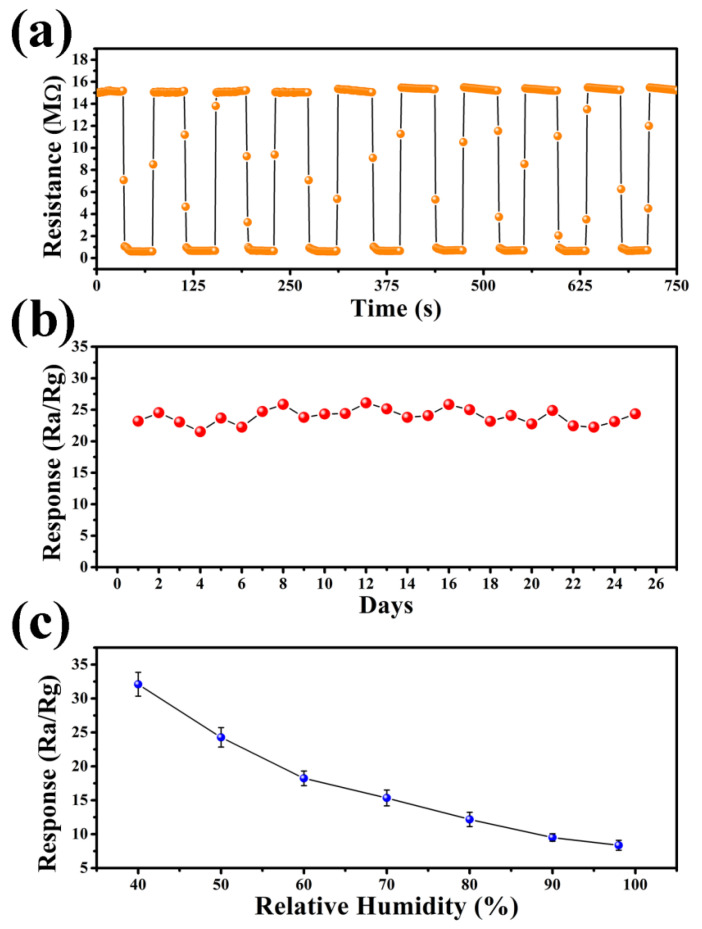
(**a**) Reproducibility, (**b**) long-term stability, and (**c**) relationship between humidity and response of the sensor based on ACS-3 to 100 ppm n-butanol at 200 °C.

**Figure 10:**
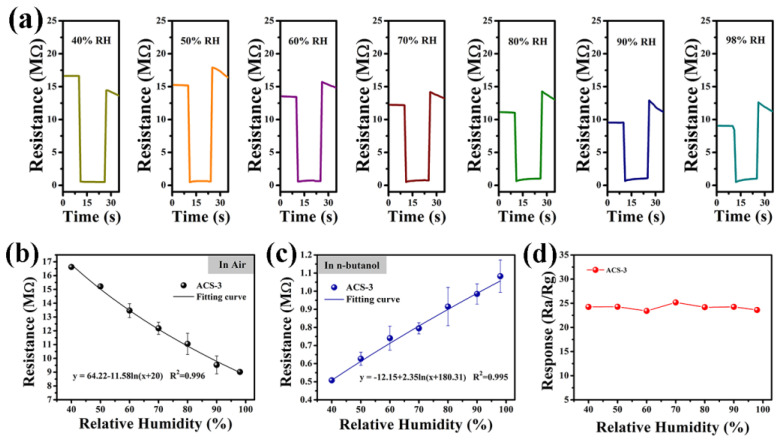
(**a**) Transient response–recovery curves to 100 ppm n-butanol, (**b**) the fitted resistance curve in air, (**c**) the fitted resistance curve in 100 ppm n-butanol, and (**d**) responses to 100 ppm n-butanol after compensation of the sensor based on ACS-3 at 200 °C under different humidity.

Figure 9a shows the dynamic response–recovery curves of ACS-3 at 200 °C for nine consecutive cycles. Throughout the successive response–recovery cycles, the sensor exhibited negligible changes in resistance in both air and test gas, with no significant decay observed in the response value, indicating excellent repeatability of the sensor. Long-term stability is crucial for practical applications. To assess this, we examined the ACS-3 sensor’s response over a period of 25 days, illustrated in Figure 9b. Throughout the uninterrupted 25-day measurement period, the sensor’s resistance consistently maintained stability at around 15 MΩ, indicating the stable properties of the sensing material under operating conditions. The impact of humidity on sensors responses were tested over the range of 40% to 98% relative humidity. The results are depicted in Figure 9c, where it is evident that the response value exhibited a significant decrease as the relative humidity increased. The sensor’s response in diverse relative humidity environments was related to the change in baseline resistance. As shown in Figure 10a, the transient curves of the sensor based on ACS-3 across different humidity levels at 200 °C showed that the baseline resistance decreased with increasing relative humidity, which can be rationalized by considering the reaction between pre-adsorbed O^−^ and water on the material surface [43,44]:

H_2_O + O^−^ = 2OH + e^−^ (2)

This reaction led to the formation of terminal hydroxyl groups and the release of an electron into the conduction band. As the baseline resistance decreased, the consumption of O^−^ reduced its reactivity with the target gas, resulting in a decline in sensor response. In addition, according to the reported literature, the resistance value of Ag_2_S is greatly affected by water molecules and can be used as a sensitive material for humidity sensors [45,46], which is also a reason why the ACS-3 sensor was significantly affected by humidity. To mitigate the effects of humidity in practical applications, humidity compensation can be implemented to minimize the disparities in sensor response under varying humidity conditions. The ACS-3 sensor’s resistance values under varying humidity levels in both air and n-butanol can be fitted using the logarithmic function, as illustrated in Figure 10b,c, with the value at 50% relative humidity as the compensation standard. The resistance compensation can be expressed by Equations (3) and (4) [47,48]:

R_a-actual_ = R_a-record_ + a1ln((H + b_1_)/(50 + b_1_)) (3)

R_g-actual_ = R_g-record_ − a2ln((H + b_2_)/(50 + b_2_)) (4)

In the equations, H denotes the relative humidity, while the correlation coefficients of the fitted curve, represented by a1, b1, a2, and b2, which are 11.58, 20, 2.35, and 180.31, respectively. The resistance values recorded in air and n-butanol are denoted as R_a-record_ and R_g-record_, while R_a-actual_ and R_g-actual_ represent the compensated resistance values. Finally, the response S_actual_ is calculated by Equation (5):

S_actual_ = R_a-actual_/R_g-actual_ (5)

As shown in Figure 10d, at relative humidity levels of 40%, 50%, 60%, 70%, 80%, 90%, and 98%, S_actual_ is 24.25, 24.29, 23.42, 25.17, 24.19, 24.26, and 23.6, respectively. The sensor with humidity compensation added maintained a stable response at various RH levels, enhancing its suitability for practical applications in the detection of n-butanol in real environments.

### 3.3. Mechanism Clarification

Gas sensors based on pristine CdS and ACS-3 exhibited rapid response and recovery to 100 ppm butanol at operating temperature. This was attributed to the one-dimensional structure of nanorod CdS, which facilitated effective electron transport along the axial direction. Additionally, in single-crystal CdS, the transport of charge carriers did not require crossing multiple potential barriers between grains, promoting the swift transmission of electrons. Both of these factors were conducive to the kinetic of the sensor.

**Figure 11:**
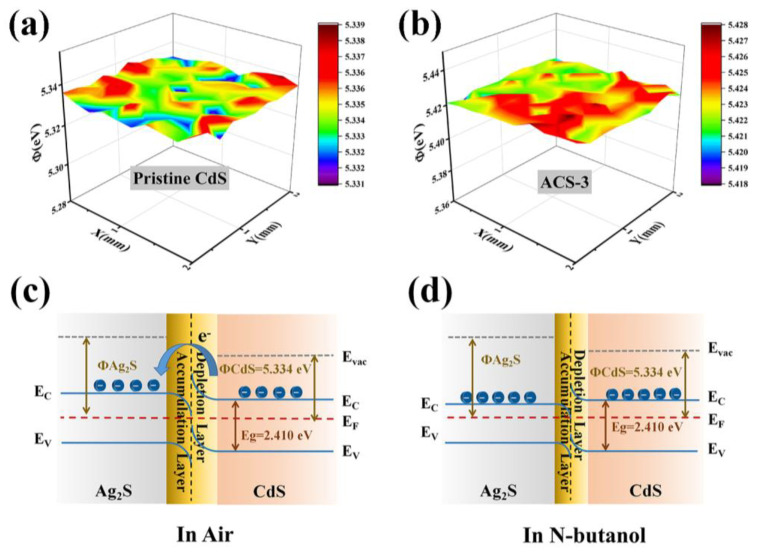
Work function of (**a**) pristine CdS and (**b**) ACS-3 measured by Kelvin probe. Schematic diagram of energy band of ACS-3 in air (**c**) and in n-butanol (**d**).

**Figure 12:**
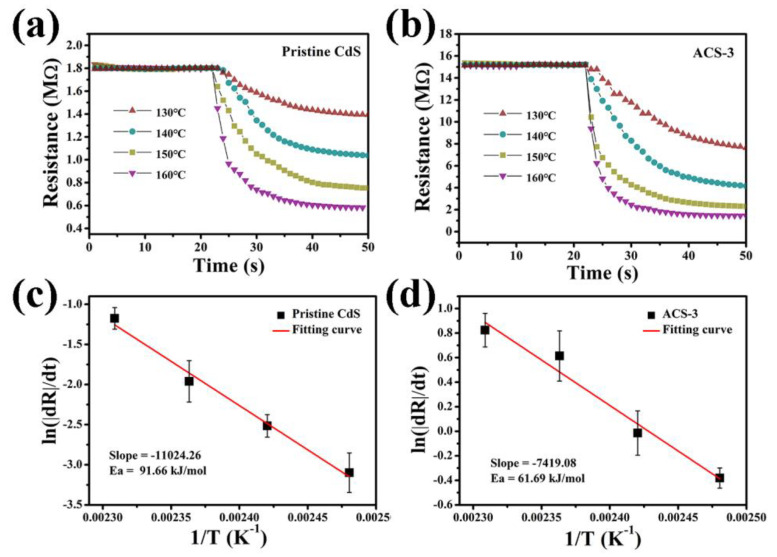
The resistance changes in the sensors based on (**a**) pristine CdS and (**b**) ACS-3 at different temperatures to 100 ppm n-butanol; Arrhenius-type plots for the rate of change in the resistance with temperature for (**c**) pristine CdS and (**d**) ACS-3 based sensors.

In comparison to sensors utilizing pristine CdS, the enhanced response of the ACS-3 sensor to n-butanol can be ascribed to two key factors:

(1) Electron sensitization: the creation of n-n heterojunction and space charge region. The KPFM results presented in Figure 11 illustrate that the work functions of pristine CdS and ACS-3 were 5.334 and 5.424 eV, respectively. There is a band gap difference between CdS and Ag_2_S, with the Fermi level of CdS being higher than that of Ag_2_S. As shown in Figure 11c, in this case, electrons will transition from the conduction band of CdS to Ag_2_S, filling the conduction band energy levels of Ag_2_S. As electrons migrate from CdS to Ag_2_S, the Fermi level gradually decreases until equilibrium is reached, resulting in a depletion layer on the surface of CdS and an accumulation layer on the surface of Ag_2_S. The excessive electron and low energy level on the side of Ag_2_S is beneficial for adsorbing more O_2_ and converting it to O^−^. During the subsequent sensing process, the reaction between n-butanol molecules and adsorbed O^−^ released electrons, disturbing the balance of n-n heterojunction, compressing the potential barrier width, and increasing the conductivity of the ACS-3 sensor. Inferred from specific surface area results, the most extensive n-n heterojunctions are present in ACS-3, which contributes to its larger resistance changes and higher sensing responses during n-butanol sensing.

(2) Chemical sensitization: Ag_2_S served as a catalyst in sensing process [49]. Overcoming the activation energy barrier was necessary for the reaction between n-butanol and adsorbed oxygen. By analyzing the resistance variations of the pristine CdS and ACS-3 sensors when exposed to n-butanol at varying temperatures facilitated the determination of the apparent activation energy (Ea), as displayed in Figure 12a,b [50]. Figure 12c,d present the Arrhenius plots of the resistance change rate (|dR|/dt) and temperature (T). The slope of the fitted relationship between ln(|dR|/dt) and 1/T was used to determine Ea. The calculated Ea values of pristine CdS and ACS-3 were 91.66 and 61.69 kJ/mol, respectively. The above results demonstrated that the incorporation of an appropriate amount of Ag_2_S reduced the activation energy of the sensing material, enabling more surface-adsorbed O^−^ to react with n-butanol gas molecules. This factor also constituted an important contributor to the heightened response of the ACS-3-based sensor towards n-butanol. Through the synergistic interplay of these two aspects, the ACS-3 sensor exhibited rapid and effective detection capabilities for n-butanol.

## 4. Conclusions

In this study, a series of CdS/Ag_2_S composite nanomaterials with varying molar ratios were synthesized using a solvothermal approach. The sensor based on the CdS/Ag_2_S composite containing 3% Ag_2_S exhibited the optimal gas sensing performance towards n-butanol. The advantageous structural characteristics of CdS single-crystal nanorods provided efficient paths for electron transport, enabling the sensor to achieve fast response and recovery rates. Leveraging the synergistic effects of electron and chemical sensitization owing to the appropriate Ag_2_S, the ACS-3 based sensor demonstrated a notable improvement in response to 100 ppm n-butanol at 200 °C, with a response value of 24.5, which was 3.9 times greater than the response of the pristine CdS sensor. Additionally, ACS-3 sensor exhibited a low detection limit of 0.5 ppm and displayed a favorable linear correlation between response and n-butanol concentration within the detection range of 0.5 to 100 ppm. The humidity compensation was employed to successfully mitigate the impact of humidity, resulting in a n-butanol gas sensor characterized by rapid response and recovery kinetics, remarkable selectivity, and long-term stability.

## Figures and Tables

**Table 1:** Comparison of n-butanol sensing ability of different gas sensors.

Sensing Materials	Operating Temperature (°C)	N-Butanol Concentration (ppm)	Response (R_g_/R_a_ or R_a_/R_g_)	Response/Recovery Time (s)	Ref.
In_2_O_3_ nanorod	240	100	342.2	77.5/34.2	[35]
Al-CdIn_2_O_4_ nanofiber	300	200	74	16/17	[1]
CdO/ZnO nanoparticle	300	200	148.64	10/31	[36]
Fe_2_O_3_/rGO nanocube	RT	200	2.71	53/42	[37]
In_2_O_3_ nanoparticle	140	50	97	45/65	[38]
Spindle Co-LaFeO_3_	320	8	52	11/8	[39]
ZnO hollow sphere	385	100	57.6	23/13	[40]
ZnSnO_3_ hollow sphere	200	50	7.1	2/40	[41]
Ni-Co_3_O_4_ nanoflower	165	100	8.4	59/63	[2]
Ag_2_O/CeO_2_/ZnO nanoflower	160	100	254	10/63	[42]
ACS-3	200	100	24.5	4/1	This work

## Data Availability

Data are contained within the article and Supplementary Materials.

## Supplementary Materials

The following supporting information can be downloaded at: https://www.mdpi.com/article/10.3390/nano14050394/s1.
