# Supplementary material for: Ag2S-Decorated One-Dimensional CdS Nanorods for Rapid Detection and Effective Discrimination of n-Butanol
Source: Nanomaterials (Basel). 2024 Feb 21;14(5):394. doi: 10.3390/nano14050394 (PMC10934644; doi:10.3390/nano14050394)
Supplement: Supplementary file 1 [file nanomaterials-14-00394-s001.zip › nanomaterials-2853698-supplementary.pdf]

Supporting Information

Ag_2S-Decorated One-Dimensional CdS Nanorods for Rapid Detection and Effective Discrimination of n-Butanol

Yubing Gao, Weirong Zhou, Yong Wang, Yuan Gao *, Jiayin Han, Dehao Kong and Geyu Lu

State Key Laboratory on Integrated Optoelectronics, Key Laboratory of Gas Sensors, College of Electronic Science and Engineering, Jilin University, 2699 Qianjin Street, Changchun 130012, China; gaoyb19@mails.jlu.edu.cn (Y.G.); zhouwr21@mails.jlu.edu.cn (W.Z.); yongw22@mails.jlu.edu.cn (Y.W.); hanjy22@mails.jlu.edu.cn (J.H.); kongdh21@mails.jlu.edu.cn (D.K.); lugy@jlu.edu.cn (G.L.)

* Correspondence: gaoyuan@jlu.edu.cn; Tel.: +86-431-85168384; Fax: +86-431-85167808

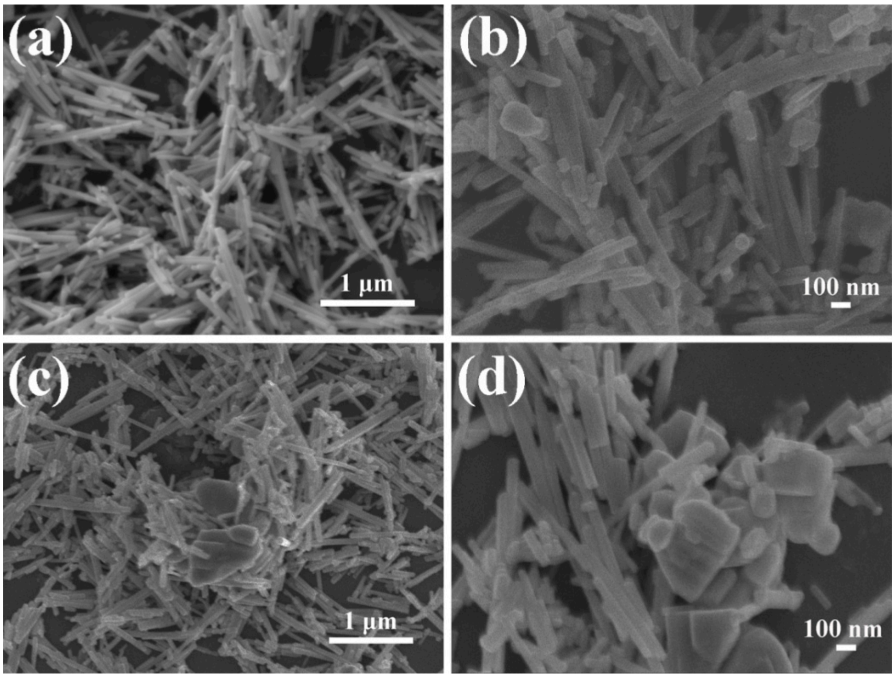

Figure S1. Low and high resolution SEM images of (a-b) ACS-1 and (c-d) ACS-5.

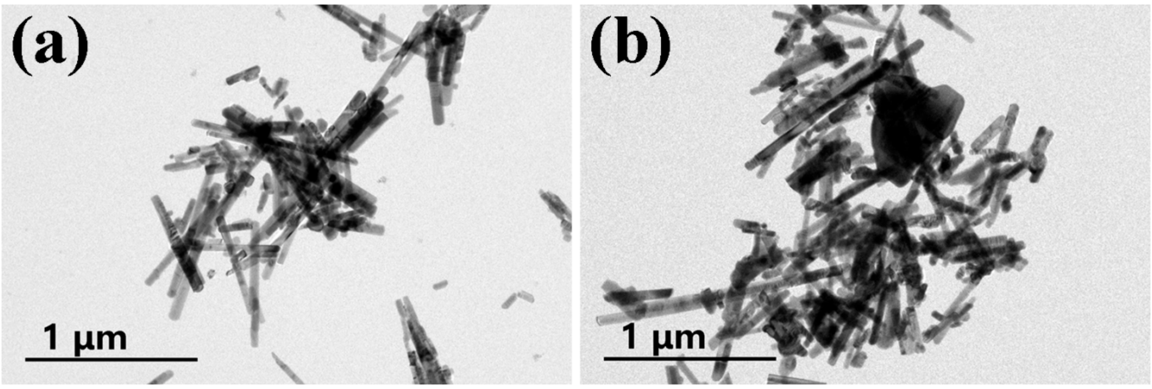

Figure S2. TEM images of (a) ACS-1 and (b) ACS-5.

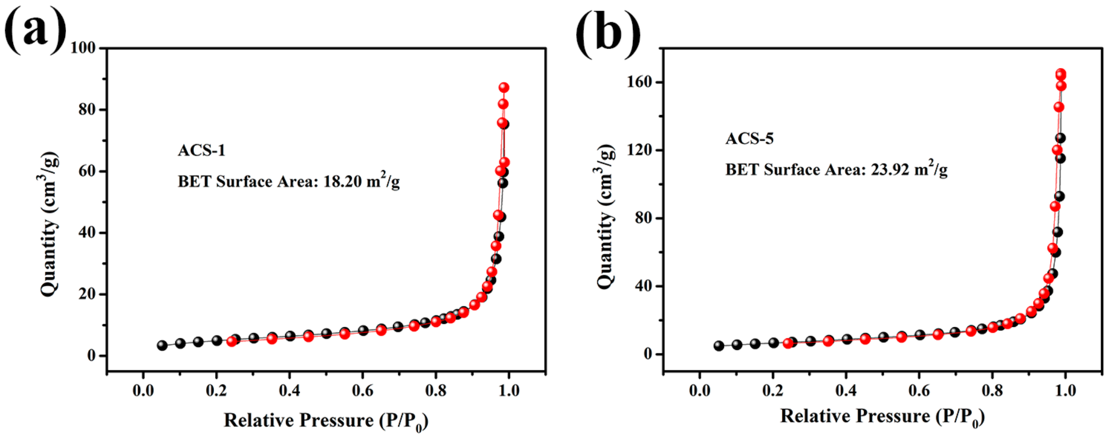

Figure S3. N_2 adsorption-desorption isotherms of (a) ACS-1 and (b) ACS-5.

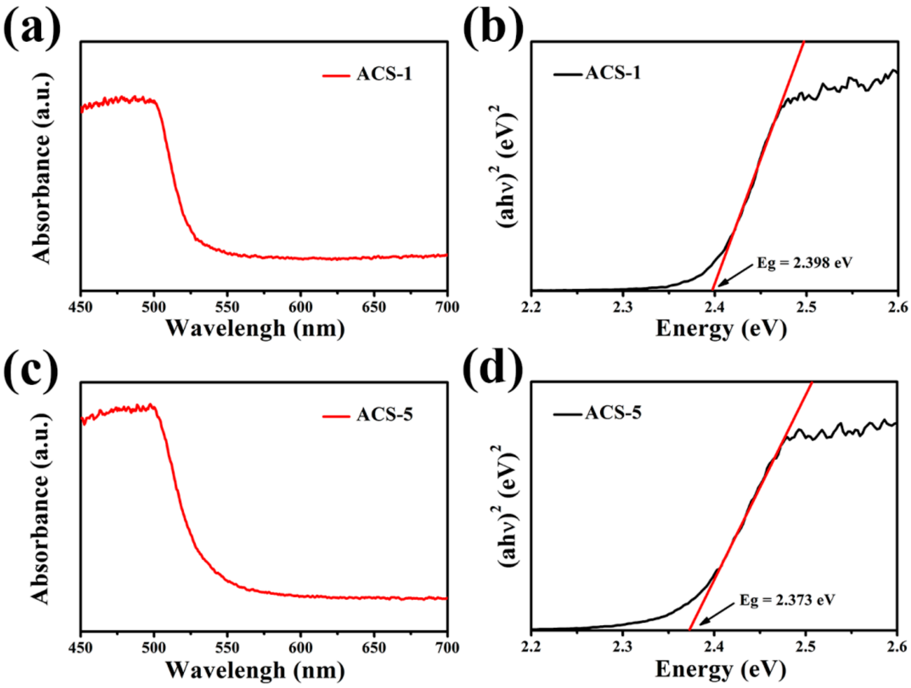

Figure S4. The UV-vis diffuse reflectance spectra of (a) ACS-1 and (c) ACS-5; Bandgap energy approximations of (b) ACS-1 and (d) ACS-5.
